# Hetero-oligomerization between the TNF receptor superfamily members CD40, Fas and TRAILR2 modulate CD40 signalling

**DOI:** 10.1038/cddis.2017.22

**Published:** 2017-02-09

**Authors:** Cristian R Smulski, Marion Decossas, Neila Chekkat, Julien Beyrath, Laure Willen, Gilles Guichard, Raquel Lorenzetti, Marta Rizzi, Hermann Eibel, Pascal Schneider, Sylvie Fournel

**Affiliations:** 1 Institut de Biologie Moléculaire et Cellulaire UPR 3572 ‘Immunopathologie et Chimie Thérapeutique' du CNRS, 15 Rue René Descartes, Strasbourg Cedex 67084, France; 2 Department of Biochemistry, University of Lausanne, Ch. des Boveresses 155, Epalinges CH-1066, Switzerland; 3 Center for Chronic Immunodeficiency, University Medical Centre Freiburg and University of Freiburg, Engesserstrasse. 4, Freiburg D-79110, Germany; 4 Institut Européen de Chimie et Biologie CBMN UMR5248 – CNRS/UB1, 2 rue Robert Escarpit, Pessac Cedex F-33607, France; 5 Department of Rheumatology and Clinical Immunology, University Medical Center Freiburg, Freiburg, Germany.

## Abstract

TNF receptor superfamily members (TNFRSF) such as CD40, Fas and TRAIL receptor 2 (TRAILR2) participate to the adaptive immune response by eliciting survival, proliferation, differentiation and/or cell death signals. The balance between these signals determines the fate of the immune response. It was previously reported that these receptors are able to self-assemble in the absence of ligand through their extracellular regions. However, the role of this oligomerization is not well understood, and none of the proposed hypotheses take into account potential hetero-association of receptors. Using CD40 as bait in a flow cytometry Förster resonance energy transfer assay, TNFRSF members with known functions in B cells were probed for interactions. Both Fas and TRAILR2 associated with CD40. Immunoprecipitation experiments confirmed the interaction of CD40 with Fas at the endogenous levels in a BJAB B-cell lymphoma cell line deficient for TRAILR2. TRAILR2-expressing BJAB cells displayed a robust CD40–TRAILR2 interaction at the expense of the CD40–Fas interaction. The same results were obtained by proximity ligation assay, using TRAILR2-positive and -negative BJAB cells and primary human B cells. Expression of the extracellular domains of Fas or TRAILR2 with a glycolipid membrane anchor specifically reduced the intrinsic signalling pathway of CD40 in 293T cells. Conversely, BJAB cells lacking endogenous Fas or TRAILR2 showed an increased NF-*κ*B response to CD40L. Finally, upregulation of TRAILR2 in primary human B cells correlated with reduced NF-*κ*B activation and reduced proliferation in response to CD40L. Altogether, these data reveal that selective interactions between different TNFRSF members may modulate ligand-induced responses upstream signalling events.

Germinal centres (GCs) are unique sites in peripheral lymphoid tissue where clonal selection of B-lymphocytes takes place in response to antigen stimulation. B-cell clonal selection results in differentiation of high-affinity B memory and antibody-secreting cells that permit an efficient secondary immune response.^1, 2, 3^ At the same time, GCs are known to be a major source of B-cell lymphomas including follicular and Burkitt's and also some diffuse large B-cell lymphoma.^4, 5^ In the process of antibody affinity maturation that takes place in GCs, B-lymphocytes with low-affinity antigen receptor are eliminated by apoptosis, whereas those with higher affinity are selected and survive. The study of how apoptotic signals synchronize in the regulation of B-cell death in GC will contribute not only to a better understanding of the mechanisms supporting clonal selection of B-lymphocytes and high-affinity antibody production but also of lymphomagenesis or progression of B lymphomas of GC origin.

Several reports showed that Fas (CD95 or TNFRSF6), a pro-apoptotic TNF receptor (TNFR) superfamily member (TNFRSF), is directly involved in the clonal selection of GC B-lymphocytes.^6, 7, 8^ Other pro-apoptotic members of the same family, TRAIL receptors are also expressed in GC B-lymphocytes.^9^ In humans, there are four membrane-bound TRAIL receptors: two death receptors TRAIL receptor 1 (TRAILR1) and TRAILR2 (DR4 or TNFRSF10A and DR5 or TNFRSF10B, respectively) that mediate death signals and two decoy receptors TRAILR3 and TRAILR4 (DcR1 or TNFRSF10C and DcR2 or TNFRSF10D, respectively). Interestingly, Fas or TRAILR-mediated apoptosis is controlled or counterbalanced in GC cells by another TNFR superfamily member CD40 (TNFRSF5).^10, 11, 12, 13^ It is conventionally accepted that CD40-induced protection against Fas or TRAILR-mediated apoptosis required NF-*κ*B activation and upregulation of anti-apoptotic c-FLIP and Bcl-X_L_ proteins.^12, 14, 15, 16^ However, Benson *et al.*^17^ described a rapid CD40-mediated rescue from Fas-induced apoptosis that did not require NF-*κ*B activation, was independent of *de novo* protein synthesis but was dependent upon active PI3K. Both previously described rescue mechanisms are ligand dependent, raising the question whether the relative amount of receptors may impact directly on CD40 signalling in a ligand-independent way.

Several TNFRSF members are able to self-associate, in particular Fas, TRAILRs and CD40.^18, 19, 20, 21, 22, 23^ We previously reported that CD40 can form non-covalent dimers in the absence of ligand, with an important contribution of the extracellular region to establish contacts.^23^ However, the potential for different TNFR family members to heteromerize has not been investigated. In this study, we identified selective interactions between CD40 and Fas, and between CD40 and TRAILR2. These heteromers also form at the endogenous level, and appear to be dynamic, driven by the preferential association of CD40 with TRAILR2 over Fas. The impact of heteromer formation on CD40 signalling was studied in cell lines and in primary human B cells, showing that they can negatively regulate CD40L-induced responses. Thus, heteromer formation between receptors with opposite functions could represent the most apical regulation of TNFRSF signalling.

## Results

### CD40 interacts with Fas and TRAILR2

**Figure 1:**
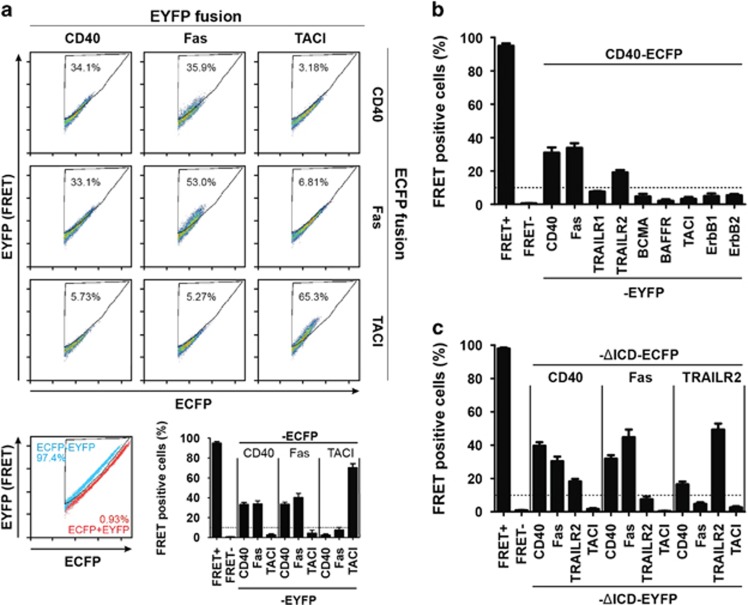
CD40 interacts with Fas and TRAILR2. (**a**) Flow cytometry FRET assay between CD40, Fas and TACI. The image shows the combination of the three receptors fused to ECFP and EYFP. The gate used to calculate the percentage of FRET-positive cells was established by using a ECFP-EYFP fusion protein (100% FRET blue dots) together with a ECFP/EYFP co-transfection (0% FRET red dots), bottom left panel. Bottom right panel shows the mean value of FRET-positive cells and SEM of five independent experiments. (**b**) Flow cytometry FRET screening for different TNFRSF members expressed in B cells. (**c**) Flow cytometry FRET assay between CD40, Fas, TRAILR2 and TACI lacking the intracellular domain (ΔICD). In all cases, FRET+ corresponds to the positive FRET reporter (ECFP–EYFP fusion protein) and FRET- corresponds to the negative control (ECFP/EYFP co-transfection). The dotted line represents background FRET levels

The first evidence of CD40–Fas interaction was obtained by Förster resonance energy transfer (FRET) by flow cytometry. Fas was initially predicted to serve as a negative control for CD40–CD40 interaction, but yielded high FRET rates when it was co-transfected with CD40 (Figure 1a). Then, we tested the ability of CD40 to interact with other TNFRSF members important for B-cell function such as Fas, TRAILR1, TRAILR2, BCMA (TNFRSF17), BAFFR (BR3 or TNFRSF13C), TACI (TNFRSF13B) and the two unrelated receptors ErbB1 and ErbB2. No interaction could be detected with ErbB1, ErbB2, TRAILR1, TACI, BAFFR or BCMA. However, positive FRET responses were observed between CD40 and Fas and, to a lesser extent, between CD40 and TRAILR2 (Figure 1b). Ligand-independent associations of CD40 with itself, with Fas and with TRAILR2 were readily observed with constructs lacking the intracellular domain (ΔICD), indicating that the latter is not required for the observed homo- and hetero-oligomerizations (Figure 1c). No interaction was detected between Fas and TRAILR2. In summary, in transiently transfected 293T cells, CD40 interacts with Fas and TRAILR2 as detected by FRET and these interactions do not require the intracellular domains.

### CD40 selectively interacts with TRAILR2 over Fas

**Figure 2:**
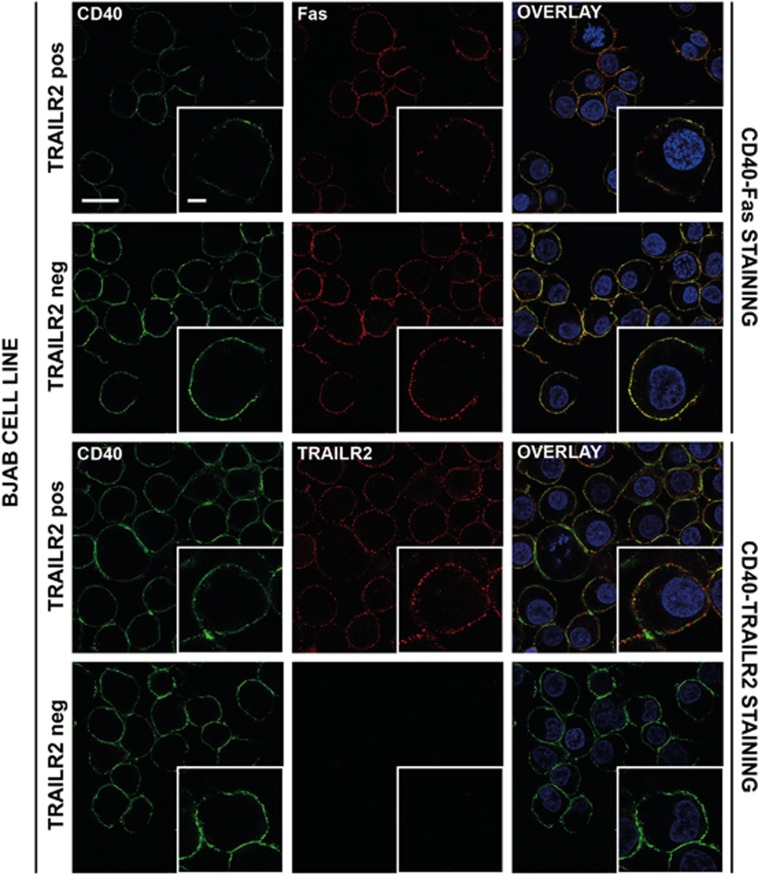
CD40, Fas and TRAILR2 colocalize at the surface of human B cells. Immunostainings of CD40-FAS and CD40-TRAILR2 in BJAB TRAILR2-positive (pos) and BJAB TRAILR2-negative (neg) cell lines. Scale bars correspond to 20 and 5 *μ*m in the main image and the inset, respectively. Fluorescence intensity plots across sections are shown in Supplementary Figures 1 and 2

In order to visualize these interactions in cells with endogenous expression levels, the CD40-positive and Fas-positive B-cell lymphoma cell line BJAB, with or without expression of TRAILR2, was used.^24^ Immunocytochemistry using antibodies against the ectodomains of CD40, Fas and TRAILR2 showed colocalization at the cell surface between CD40 and Fas in TRAILR2-negative BJAB cells. However, colocalization of CD40 and Fas was reduced in TRAILR2-positive BJAB when compared with TRAILR2-negative BJAB cells, despite similar Fas expression levels. Under the same conditions, colocalization of CD40 and TRAILR2 was detected in TRAILR2-positive BJAB cells (Figure 2, Supplementary Figures 1 and 2).

**Figure 3:**
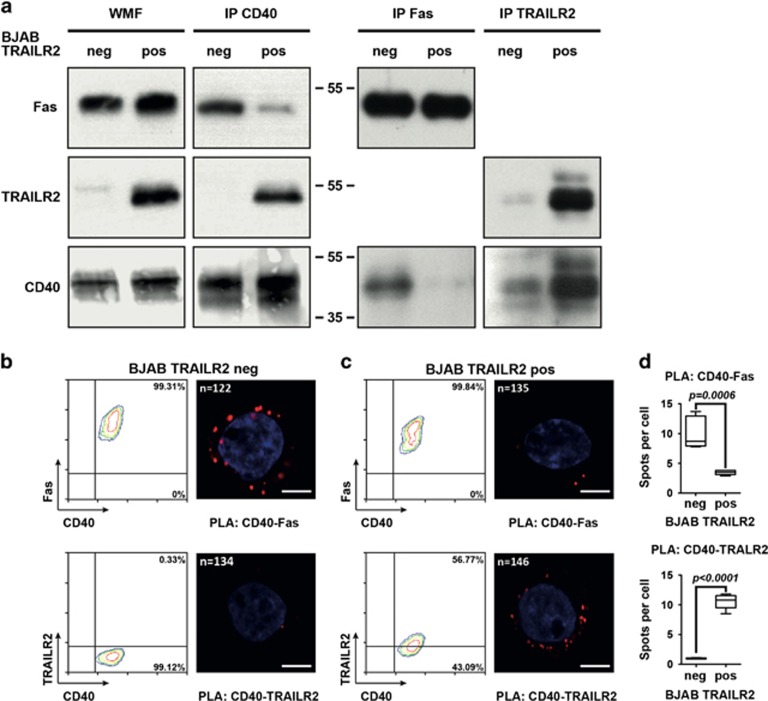
CD40 selectively interacts with TRAILR2 over Fas. (**a**) Co-immunoprecipitation of CD40, Fas and TRAILR2 in BJAB TRAILR2-negative (neg) and TRAILR2-positive (pos) cells followed by WB detection of CD40, Fas and TRAILR2. (WMF: input of whole membrane fraction). (**b**) FACS expression profile of CD40 and Fas or CD40 and TRAILR2 on BJAB cell line TRAILR2-negative (left panel) together with a confocal image of the same cell type analyzed by PLA (right panel) for both CD40-Fas or CD40-TRAILR2 interactions. (**c**) Same as panel B, for the TRAILR2-positive BJAB cell line. (**d**) Mean and S.E.M. of the PLA assay. Spot numbers per cell were counted in the focal plane. The number of cells analyzed by PLA is indicated on each image in panels **b** and **c**. Scale bars in panels **c** and **d** correspond to 10 *μ*m

In line with these results, we found that CD40 and Fas co-immunoprecipitated in native membrane fractions of TRAILR2-negative BJAB cells, but that this interaction was strongly decreased in TRAILR2-positive cells, which instead showed co-immunoprecipitation of CD40 with TRAILR2 (Figure 3a). We next assessed these interactions by means of proximity ligation assays (PLAs),^25^ using the same primary antibodies used for immunocytochemistry. This technique allows the detection of interacting proteins at endogenous levels by connecting two protein-specific antibodies in close proximity with a complementary DNA probe that is annealed, amplified and visualized as a fluorescent spot. We found CD40-Fas spots on TRAILR2-negative BJAB cells and, as expected, only background CD40-TRAILR2 spots (Figures 3b and d). Interestingly, the number of CD40-Fas spots was significantly diminished in TRAILR2-positive BJAB cells, with a concomitant appearance of CD40-TRAILR2 spots (Figures 3c and d). These results, together with those of the co-immunoprecipitation experiments, indicate that these interactions can be found at the endogenous levels, and that CD40 selectively associates with TRAILR2 over Fas in BJAB cells.

### CD40 interacts with Fas and TRAILR2 in primary human B cells

**Figure 4:**
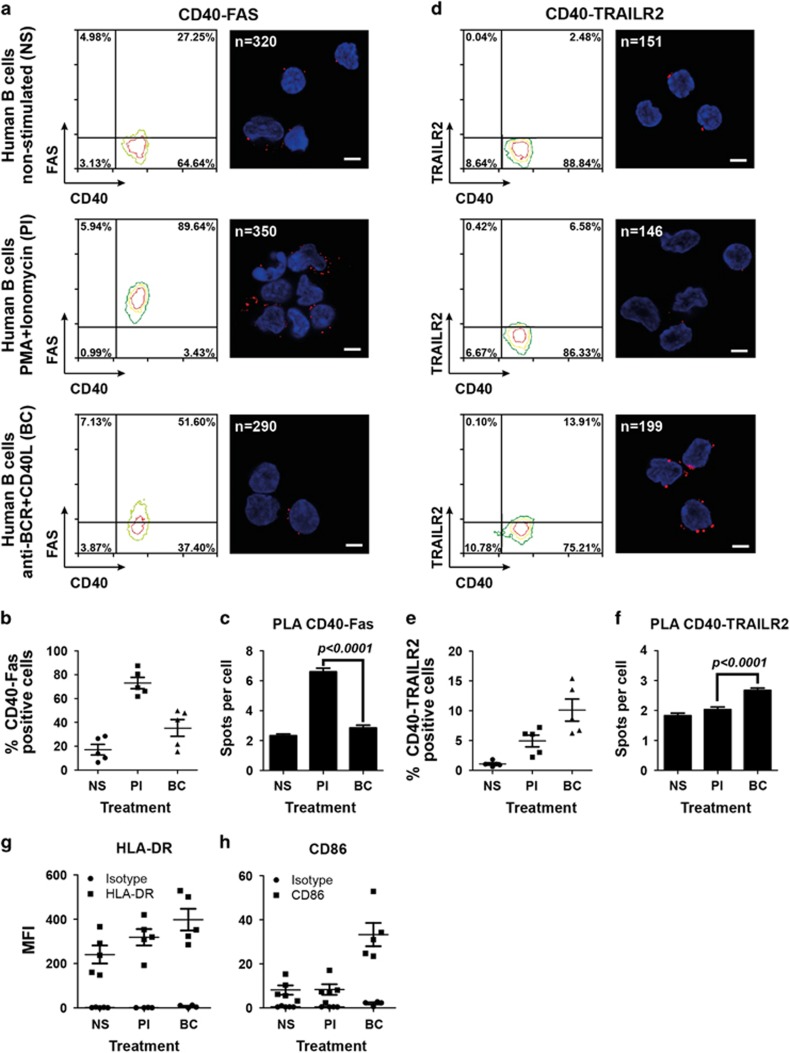
CD40 interacts with Fas and TRAILR2 in primary human B cells. (**a**) FACS expression profile of CD40 and Fas on primary human B cells non stimulated (NS), activated with PMA-ionomycin (PI) or activated with anti-BCR plus CD40L (BC) (left panels) together with a confocal image of the same cell type analyzed by PLA for CD40-Fas interaction (right panels) (one representative image out of three independent donors is shown). (**b**) Percentage of CD40-Fas double-positive cells in five different donors treated as described in panel **a**. (**c**) Mean and S.E.M. of the PLA assay for the three donors analyzed by PLA. Spot numbers were counted in positive cells in the focal plane. (**d, e** and **f**) Same as **a**–**c**, but for CD40–TRAILR2 interactions. The number of cells analyzed in the example is indicated on each image. Scale bars correspond to 5 *μ*m. (**g**) HLA-DR and isotype control mean fluorescence intensity (MFI) and S.E.M. of five different human primary B cells samples that were either NS, activated with PI or activated with anti-BCR plus CD40L (BC). (**h**) Same as **g**, but for CD86 detection

To investigate whether CD40 associates with Fas and TRAILR2 in primary human B cells, peripheral B-lymphocytes were isolated and activated with two different protocols. Without stimulation, these cells express high levels of CD40, low levels of Fas and no detectable TRAILR2 (Figure 4a). Treatment with PMA and ionomycin induced a mild activation profile characterized by increased MHC II expression but little changes in CD86 levels, associated with a strong upregulation of Fas but not TRAILR2 (Figures 4a, b, g and h). In contrast, treatment with an anti-BCR antibody (anti-IgM/IgG) plus CD40L induced a strong activation profile characterized by increased levels of both MHC II and CD86, associated with a mild increase of Fas and TRAILR2 levels (Figures 4d, e, g and h). These stimulation protocols somewhat reproduce the situation of TRAILR2-positive and -negative BJAB cells, although the expression levels of Fas were decreased when TRAILR2 levels were increased and, consequently, the percentage of CD40 and Fas double-positive cells was reduced when the percentage of CD40 and TRAILR2-positive cells was increased (Figures 4b and e). PLA assays on activated human B cells detected the interaction of CD40 with Fas in the absence of TRAILR2 (PMA/ionomycin activation). This interaction was strongly reduced when less Fas was expressed (BCR/CD40L activation); it is possible that the concomitant increase in TRAILR2 expression may contribute to further reduce this signal (Figures 4a and c). In line with the low expression of TRAILR2 after PMA/ionomycin activation, little CD40-TRAILR2 spots were present in this condition but a slight, yet significant increase of CD40-TRAILR2 spots was observed when a modest upregulation of TRAILR2 was achieved by stimulation with BCR/CD40L (Figures 4d and f). Similar PLA results were obtained with B cells of three different donors (Figures 4g and h). These results confirm the interaction of CD40 with Fas or with TRAILR2 on activated human B-lymphocytes.

### Signalling-incompetent TRAILR2 or Fas decreases CD40 signalling

**Figure 5:**
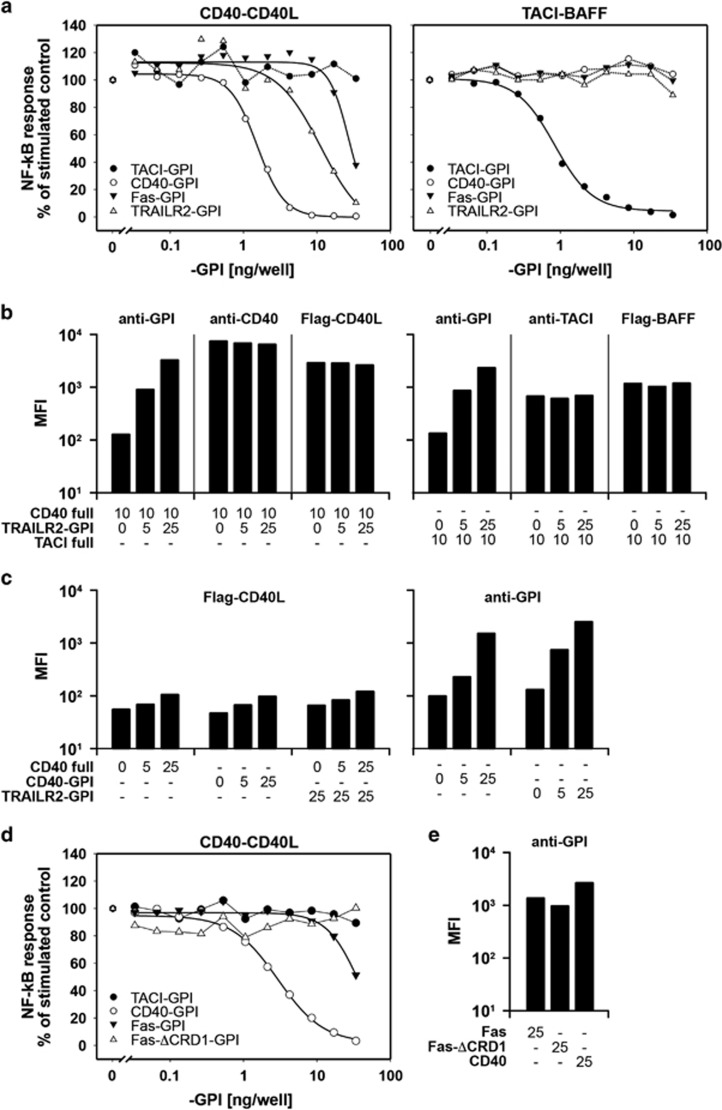
Heteromer formation with signalling-incompetent TRAILR2 or Fas has a negative impact on CD40 signalling. (**a**) NF-*κ*B luciferase assay in 293T cells transiently transfected with 0.5 ng of CD40, 1 ng of CD40L (left panel) or with 0.5 ng of TACI, 1 ng of BAFF (right panel) and increasing amounts of GPI-anchored CD40, TRAILR2, Fas or TACI. One out of three independent assays with similar results is shown. (**b**) Flow cytometry analysis of TRAILR2-GPI, CD40 full-length and TACI full-length surface expression. (**c**) Left: surface expression levels of CD40 full-length, CD40-GPI and CD40 full-length in the presence of TRAILR2, detected by staining with Flag-CD40L. Right, surface expression levels of CD40-GPI and TRAILR2-GPI, detected by staining with an anti-TRAILR3 mAb recognizing the GPI-proximal region of the fusion receptors. MFI, mean of fluorescence intensity. (**d**) NF-*κ*B luciferase assay in 293T cells transiently transfected with 0.5 ng of CD40, 1 ng of CD40L and increasing amounts of GPI-anchored Fas, Fas-ΔCRD1, CD40 and TACI. One out of two independent assays with similar results is shown. (**e**) Flow cytometry analysis of Fas and Fas-ΔCRD1 surface expression

We wanted to determine whether heteromerization of CD40 with TRAILR2 or Fas could modulate CD40-mediated NF-*κ*B activation. However, Fas and TRAILR2 can also induce NF-*κ*B activation^26^ and overexpression of Fas or TRAILR2 may indirectly impact on CD40 signals by inducing cell death. To rule out any direct contribution of Fas and TRAILR2 to NF-*κ*B signalling, we used glycolipid-anchored constructs of these receptors lacking transmembrane and intracellular domains,^27^ and observed how these truncated receptors may change CD40-mediated signals. 293T cells were transfected with a constant amount of CD40 and CD40L together with increasing concentrations of glycosyl-phosphatidylinositol (GPI)-anchored CD40, TRAILR2, Fas or TACI ectodomains, and the NF-*κ*B response was quantified using a luciferase reporter. As expected, increasing concentrations of CD40-GPI inhibited CD40 signalling, most probably by competition for CD40L binding. However, increasing concentrations of TRAILR2-GPI, which is unable to compete for CD40L binding, also inhibited CD40 signalling. A similar result was observed with higher amounts of Fas-GPI but no impact in CD40 signalling was observed upon expression of TACI-GPI (Figure 5a, left). None of these receptors (CD40-, Fas- or TRAILR2-GPI) had an effect on TACI-induced NF-*κ*B response, indicating a specific impact on CD40 signalling (Figure 5a, right). The amount of transfected plasmid sufficient to inhibit 50% of CD40-induced NF-*κ*B response was about 1.4 ng of CD40-GPI for 0.5 ng of full-length CD40. For TRAILR2-GPI, the amount of transfected plasmid sufficient to inhibit 50% of CD40-induced NF-*κ*B response was about 11 ng per well. The differences between the strength of TRAILR2-GPI and Fas-GPI-mediated inhibition could be explained either by the endogenous expression of TRAILR2 in 293T cells, or by differences in the interaction affinities with CD40. The expression levels of TRAILR2-GPI remained relatively constant in the presence of either CD40 or TACI. Similarly, expression levels of CD40 and TACI were insensitive to the presence of TRAILR2-GPI (Figure 5b). In addition, TRAILR2-GPI did not impact on CD40–CD40L interaction, as determined by flow cytometry staining using Flag-tagged CD40 ligand (Figure 5b). Full-length CD40 and CD40-GPI showed similar expression levels on the cell surface when the same amount of plasmid was transfected (Figure 5c). Finally, GPI-anchored receptors were all expressed at similar levels (Figure 5c).

It was shown that the cysteine-rich domain 1 (CRD1) of Fas,^18^ TRAILR2^20^ and CD40^23^ mediates receptor self-assembly. In contrast to Fas-GPI, Fas-ΔCRD1-GPI did not interfere with CD40-mediated NF-*κ*B activation, suggesting that the CRD1 of Fas may be involved in Fas-CD40 interaction (Figure 5d). No conclusive results could, however, be obtained with TRAILR2-ΔCRD1-GPI because this protein did not reach the cell surface, most probably due to folding problems similar to those reported when the CRD1 of CD40 was deleted or modified.^23^ Taken together, these results indicate that the extracellular domains of Fas and TRAILR2, but not that of TACI, negatively affect CD40L-mediated signalling in the absence of any direct contribution of Fas or TRAILR2 to the signal or detectable changes in the levels of CD40 expression or its ability to interact with CD40L.

### Endogenous TRAILR2 or Fas modulate CD40 signalling

**Figure 6:**
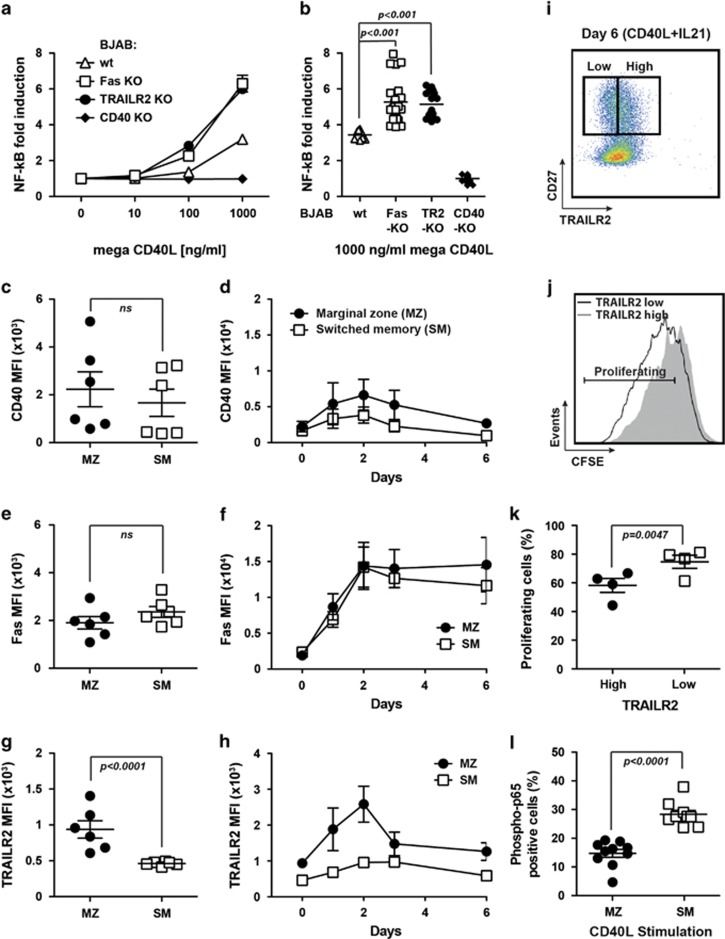
Endogenous TRAILR2 or Fas modulate CD40 signalling. (**a**) NF-κB response of wild-type, Fas KO, TRAILR2 KO and CD40 KO BJAB cell lines induced by the indicated concentrations of Flag-ACRP-CD40L (mega-CD40L). One out of two independent experiments is shown (**b**). Average of the different clones and replicates of each KO cell line stimulated with 1 *μ*g/ml of mega-CD40L. (**c**) *Ex vivo* expression profile of CD40 in marginal zone (MZ) and switched memory (SM) primary human B cells. (**d**) Expression profile of CD40 in MZ and SM along 6 days of CD40L+IL-21 stimulation. (**e** and **f**) Same as **c** and **d**, but for Fas expression. (**g** and **h**) Same as **c** and **d**, but for TRAILR2 expression. MZ B cells are gated as CD27+, IgD+ and SM B cells are gated as CD27+ IgD-. The mean and S.E.M. of six independent experiments is shown. (**i**) TRAILR2 expression in CD27+ B cells of one representative donor out of four tested after six days of stimulation with CD40L+IL-21. The same gating was used in panel **j**. (**j**) CFSE proliferation profile of TRAILR2 low CD27+ (grey histogram) and TRAILR2 high CD27+ (black line) primary human B cells of one representative donor out of four tested after six days of stimulation with CD40L+IL-21. (**k**) Average proliferative response for the four independent donors analyzed. (**l**) Flow cytometry analysis of phospho-p65 (RelA) after 5-min stimulation with CD40L. The figure shows five independent donors analyzed in duplicate

To address the impact of endogenous Fas and TRAILR2 on CD40 signalling, we generated several clones of BJAB cells knocked-out for each of these receptors using the CRISPR/Cas9 system^28^ (Supplementary Figure 3). When tested for their ability to respond to CD40L, all Fas and TRAILR2 KO clones showed a significant, dose-dependent increase in CD40L-induced NF-*κ*B response when compared with wild-type cells, whereas CD40 KO BJAB clones were, as expected, not responsive to CD40L stimulation (Figures 6a and b). These changes in the NF-*κ*B response strongly suggest that CD40-Fas and CD40-TRAILR2 heteromerization regulates CD40 signalling, even if it is difficult to exclude that the absence of Fas or TRAILR2 may indirectly modulate this outcome.

In primary human B cells, the expression profile and the balance between CD40 and Fas signalling has been well described,^1^ however, the expression and function of TRAILR2 remains poorly explored in B-cell subsets. We found that primary human marginal zone B cells (MZ: CD27+ IgD+) express higher levels of TRAILR2 compared with switched memory cells (SM: CD27+ IgD-), whereas the expression of CD40 and Fas was similar in these two B-cell populations (Figures 6c, e and g). This difference was enhanced after stimulation with CD40L+IL-21, with a peak of TRAILR2, Fas and CD40 expression at day 2 (Figures 6d, f and h). To study the impact of TRAILR2 expression on CD40 activation, we used CFSE-labelled cells to analyze the proliferative response of CD27+ B cells to CD40L stimulation. After 6 days of activation, TRAILR2 high cells did not proliferate as strongly as TRAILR2 low cells. A similar response was observed in four independent donors (Figures 6i and k). Phosphorylation of the NF-*κ*B subunit p65/RelA was measured by flow cytometry as a marker of NF-*κ*B activation in response to stimulation with CD40L. The percentage of phospho-p65-positive cells was significant higher in switched memory cells (that have lower levels of TRAILR2) than in marginal zone B cells (that have higher levels of TRAILR2) (Figure 6l). Taken together, these results suggest that endogenous TRAILR2 may increase the threshold required to obtain CD40L-induced responses in cell lines and in primary human B cells.

## Discussion

There are increasing evidences suggesting that TNFR superfamily members are organized in the cell membrane as ligand-independent oligomers rather than as individual receptors. Different receptors of the family have been described forming homo-dimers or -trimers, such as TNFR1 and 2,^19^ Fas,^18^ TRAIL receptors,^20^ CD40^23^ and BAFFR.^29^ This pre-ligand assembly was reported in most cases to favour ligand binding and normal signalling.^18, 19, 20, 29^ However, the potential heteromerization of TNFRs remains poorly explored and there are only three studies addressing this phenomenon, one between TRAILR2 and TRAILR4^20^ and two others in the context of the central nervous system that describe the interaction of DR6 with p75^NTR^^30^ and DR6 with TROY.^31^ From the screening performed by flow cytometry FRET using CD40 as bait, we consistently found high FRET rates with Fas and to a lesser extent with TRAILR2. Other receptors important for B-cell survival and differentiation were negative in this screening. These interactions did not require the presence of the intracellular domain, which is in line with the fact that most of the TNFRSF–TNFRSF interactions described to date rely on the extracellular region of the receptors.

CD40-Fas and CD40-TRAILR2 interactions take place at the endogenous levels in the Burkitt B-cell lymphoma BJAB cell line expressing or not TRAILR2. Interestingly, the CD40–Fas interaction was only detected in TRAILR2-negative BJAB cells despite similar expression levels of CD40 and Fas in both cell lines, indicating a possible competition between TRAILR2 and Fas for CD40 binding. This competition at the endogenous levels was evidenced by co-immunoprecipitations and by PLAs. However, the molecular basis for the observed selectivity in these interactions remains unclear. CD40 may have a higher affinity for TRAILR2 than for Fas, but these interactions may also be influenced by the relative abundance of each receptor on the cell surface. Finally, we cannot exclude that Fas or TRAILR2 may localize to specialized membrane micro-domains in which CD40 could be recruited.

PLA studies confirmed that both CD40-Fas and CD40-TRAILR2 are close together in primary human B cells. In these experiments, CD40 was expressed in all conditions, and it was possible to preferentially induce Fas or TRAILR2 using different activation protocols. Cells in both stimulation conditions were not exactly comparable: Fas expression levels were different, and CD40 was engaged by ligand in one but not the other condition. Nonetheless, the results are fully compatible with a model in which CD40 preferentially interacts with TRAILR2 rather than with Fas. The preferred interaction between CD40 and TRAILR2 may thus be common to lymphoma cell lines and primary cells.

We have shown that expression of signalling-incompetent TRAILR2 or Fas, unable to interact with CD40L, decreased or sometimes totally abolished the ability of full-length CD40 to respond to CD40L with NF-κB activation. Under these conditions, surface expression of CD40, and its ability to bind CD40L were unaffected, indicating that heteromers interfere with the signalling function by acting downstream of ligand binding, probably by interfering with formation of a functional signalling complex. Surface expression of full-length and GPI-anchored CD40 was similar, allowing comparisons to be performed. At the amount of transfected plasmid DNA sufficient for a 50% inhibition of CD40-induced NF-*κ*B response, CD40-GPI was in a 3-fold excess over full-length CD40, and TRAILR2-GPI was in a 20-fold excess, which appears to be within a physiological range, especially when considering that the GPI fusion receptors may not be optimal for heteromer formation. In the case of TRAILR2-GPI, we carefully excluded the possibility of artefactual quenching of CD40L or CD40, suggesting that the observed effects are due to heteromer formation. It is tempting to speculate that the combined outcome of signalling via CD40 on the one hand, and TRAILR2 and Fas on the other hand, is not only regulated intracellularly by activation of pro- or anti-apoptotic signalling pathways, but also directly at the level of receptors whose relative expression levels may determine whether they can be activated or not. For several TNFRs, a pre-ligand assembly domain (PLAD) has been described that mediates homo-interactions.^18, 19, 20, 23^ The deletion of the CRD1 of Fas was sufficient to abrogate its inhibitory effect on CD40 signalling indicating that the PLAD of Fas may have a role in heteromer formation. However, TRAILR2 with a similar deletion did not reach the cell surface, precluding interpretation of whether TRAILR2 CRD1 is involved or not in heteromer interactions. The inhibitory impact of Fas and TRAILR2 on CD40 signalling has been confirmed in Fas and TRAILR2 KO BJAB cell lines, which showed increased responsiveness to CD40L.

In the biology of B cells, Fas and TRAILR2 are death receptors that trigger apoptosis of autoreactive and/or activated B cells,^32, 33^ whereas CD40 is a potent NF-*κ*B activator that provides activation and proliferation signals.^34, 35^ However, the interplay between these three receptors is complicated and not fully understood. Several studies focused on the role of Fas during high-affinity B-cell selection in GC showing that Fas has an essential role in GC B-cell apoptosis both *in vitro*^36^ and *in vivo*.^6^ During B-cell maturation, somatic hypermutations are introduced in the variable regions of heavy and light chains of the BCR with the aim of generating antigen-specific B cells of higher affinity.^37^ A stringent selection mechanism takes place to ensure the survival of antigen-specific high-affinity B cells and the death of low-affinity or autoreactive B cells. GC B cells express CD40 and Fas and undergo FasL-mediated apoptosis, unless a survival signal is provided by BCR or CD40 engagement.^38, 39, 40, 41^ The same type of data does not exist for TRAILR2, but it was shown *ex vivo* on primary human B cells that BCR and/or CD40L signals can rescue naive B cells, but not memory B cells, from TRAIL-induced apoptosis.^13^ Together, these data suggest that signals transduced by Fas, TRAILR2 and CD40 are entangled to finely control the fate of B cells. In B-cell lymphomas, the relationship between CD40, Fas and TRAILRs appears even more complicated. As in normal GC B-lymphocytes, CD40 rescues apoptosis induced by Fas in low-grade B lymphoma, but CD40 sensitizes Burkitt lymphoma B cells to Fas-induced apoptosis.^42, 43, 44^ In a similar way, CD40 triggering protects Burkitt lymphoma^13^ and follicular lymphoma,^45, 46^ but sensitizes chronic lymphocytic leukaemia B cells to apoptosis induced by TRAIL.^47^ The measure of TRAILR2 expression revealed that expression of this receptor is increased in marginal zone compared with switched memory B cells and that this difference is maintained upon CD40L+IL-21 stimulation. Our *in vitro* data would be in line with the notion that TRAILR2 negatively regulates CD40L effects, as its expression inversely correlated with CD40L-induced proliferation in primary human B cells. Accordingly, CD40L-induced phosphorylation of p65 (RelA) inversely correlated with TRAILR2 expression in primary human B cells. This was true when analyzing percentage of phospho-p65-positive cells, but was not as marked when analyzing mean fluorescence intensities, suggesting that TRAILR2 may increase the threshold of CD40L stimulation required to activate CD40, but may not modify signalling once CD40 has been activated.

At this stage of the study, experiments performed in primary cells correlate TRAILR2 expression with lower responses to CD40L (NF-*κ*B and proliferation). It would be interesting in the future to knock-out TRAILR2 from primary human B cells and test whether this increases responsiveness to CD40L, or to produce a mouse model with inducible knock-out of TRAILR. Mice, however, display significant differences with human in their array of TRAIL receptors,^48^ and it would be necessary to test first whether TRAILR–CD40 interactions also take place in mice.

In conclusion, our results reveal that ligand-independent heteromer formation between different TNFRSF members may be involved in the modulation of very early steps of activation, upstream of their signalling pathways.

## Materials and methods

### Cell lines

BJAB cells expressing TRAILR2 or not were grown in RPMI 1640 medium (Lonza, Verviers, Belgium) supplemented with 10% foetal calf serum and 5 *μ*g/ml each of penicillin and streptomycin. HEK 293T cells were grown in DMEM medium (Gibco, Carlsbad, CA, USA) supplemented with 10% foetal calf serum. BJAB Fas KO, BJAB TRAILR2 KO and BJAB CD40 KO were generated by lentiviral transduction using CRISPR/Cas9 expression vectors carrying the corresponding gRNAs (Supplementary Table S1), according to Shalem *et al.*^28^

### Antibodies and TNF ligands

Western blot: anti-CD40 S-17 (Santa Cruz Biotechnology, Dallas, TX, USA), anti-Fas (ZB4) (Abcam, Cambridge, UK), anti-DR5 (Millipore, Billerica, MA, USA). Immunoprecipitations: anti-CD40 C20 agarose conjugate (Santa Cruz Biotechnology), anti-Fas (C20) (Santa Cruz Biotechnology), anti-DR5 D4E9 (Cell Signalling, Danvers, MA, USA). Flow cytometry: anti-CD40-FITC and –PE-Cy5 (5C3), anti-Fas-PE (DX2), anti-MHC-II-FITC (Tü 39), anti-CD86-PE (2331) and anti-CD19-V500 (HIB19) (BD Pharmingen, San Diego, CA, USA); anti-CD27-BV421, anti-CD95-AF647 and anti-IgD-PE-Cy7 (Biolegend, San Diego, CA, USA); anti-DR5-PE (B-K29) (Diaclone Research, San Diego, CA, USA) for cell lines; anti-TRAILR2 PE (eBioscience, San Diego, CA, USA) for primary cells; anti-TRAILR2 (TR2.21; to screen TRAILR2 KO BJAB clones) (Adipogen, Lausen, Switzerland); anti-phospho-NF-*κ*B p65 (Ser536, clone 93H1)-AF647 (Cell Signalling). Flag-ACRP-hBAFF, Flag-ACRP-hCD40L and Flag-ACRP-FasL were produced in house and staining were performed as described in Bossen *et al.*^27^ Flag-ACRP-hCD40L (mega-CD40L) was obtained from Adipogen. Anti-TRAILR3 (LEIA) was used for GPI recognition (own production).^27^ Microscopy and PLA: monoclonal rabbit anti-CD40 (EBI-19-21) (Abcam), mouse anti-DR5 (DJR2-4) (ABD Serotec, Raleigh, NC, USA) and mouse anti-CD95 (DX2) (BD Biosciences, San Diego, CA, USA).

### Expression plasmids

See Supplementary Table 1.

### Förster resonance energy transfer

Experiments were performed using a LSRII flow cytometer instrument (BD Biosciences). EYFP signal was recorded using the 488 nm laser with a 530/30 filter, ECFP signal was recorded using the 405 nm laser with a 450/50 filter and FRET signal was recorded using the 405 nm laser with a 585/42 filter. For each condition, we evaluated a minimum of one thousand -ECFP -EYFP double-positive cells. HEK 293T cells were transiently transfected with ECFP and EYFP fusion receptors and analyzed 16–20 h post-transfection. Positive FRET cells were gated using an ECFP–EYFP fusion protein as positive control and a co-transfection of ECFP and EYFP as negative control according to Banning *et al.*^49^ and Schneider *et al.*^50^

### Immunocytochemistry

BJAB cells grown on Lab-tek chambers were fixed with 2% formaldehyde and incubated with primary antibodies overnight at 4 °C (rabbit anti-CD40 50 *μ*g/ml, mouse anti-TRAILR2 25 *μ*g/ml or biotinylated mouse anti-Fas 1/10) in 1% normal goat serum. Cells were then incubated for 1 h with F(ab)'2 goat anti-rabbit-A488 (1/200) plus mouse-A594 (1/200) or in F(ab)'2 goat anti-rabbit-A488 (1/200) plus streptavidin A546 (1/1000) for the detection of CD40-TRAILR2 or CD40-Fas, respectively. After post-fixation in 4% formaldehyde, nuclei were counterstained with DAPI (1/500). Slides were then mounted with Dako fluorescent medium (Glostrup, Denmark) and images were acquired using a LSM 510 Laser Scanning Microscope (Carl Zeiss, Jena, Germany).

### Immunoprecipitations

In all, 1 × 10^7^ TRAILR2-negative and TRAILR2-positive BJAB cells were harvested and the native membrane fraction was obtained using ProteoExtract kit (Calbiochem, San Diego, CA, USA). Immunoprecipitations were performed with agarose-conjugated anti-CD40 antibody or protein A-loaded antibodies (Fas and TRAILR2) as described in Schneider *et al.*^50^ Immunoprecipitates and whole membrane fractions were resolved on 10% acrylamide SDS gels before electrophoretic transfer onto nitrocellulose and western blot analysis.

### Duolink *in situ* PLA

PLA analysis was performed according to the manufacturer's instructions (OLink Biosciences, Uppsala, Sweden). Briefly, cells were incubated for 1 h at 37 °C on poly-lysine coated slides, fixed with 2% formaldehyde and blocked with 2% of normal donkey serum. Primary antibodies (used at the same dilutions than for immunocytochemistry) were incubated overnight at 4 °C, cells were then incubated with PLUS and MINUS secondary PLA probes against both rabbit and mouse IgG. Hybridization and ligation steps followed the incubation, and then amplification was performed. After mounting with Duolink mounting medium, images were acquired using a LSM 700 Laser Scanning Microscope in the larger focal plane of each cell (Carl Zeiss). Quantification was done using ImageJ.^51^

### Human B-cell purification and culture

Peripheral blood mononuclear cells were first isolated using Ficoll (Sigma, St. Louis, MO, USA). Then, B-lymphocytes were purified by negative magnetic sorting (MACS) using the human B-cell purification kit from Miltenyi Biotec (Paris, France). Cells were then incubated in RPMI-1640 supplemented with 10% foetal calf serum and 5 *μ*g/ml each of penicillin and streptomycin for 24 h and treated as follows: 50 ng/ml PMA (phorbol-myristate-acetate) (Sigma-Aldrich, St. Louis, MO, USA) and 1 mM ionomycin (Sigma-Aldrich) or 0.5 ng/ml hCD40L-muCD8 (Ancell, Bayport, MN, USA) and 20 *μ*g/ml of anti-IgM/IgG (Sigma-Aldrich). Cells were then subjected to flow cytometry and PLA analysis. For CD40, Fas and TRAILR2 expression analysis, MACS sorted B cells (1.5 × 10^5^ cells/ml) were stimulated with CD40L and IL-21 in enriched Iscovés medium supplemented with 10% FCS, 1 *μ*g/ml insulin, 2.5 *μ*g/ml apo-transferrin, 0.1% fatty acid supplement, 1% non-essential amino acids, 2 mM glutamine and 1 *μ*g/ml reduced glutathione as described.^52^ B cells were stained and analyzed by flow cytometry at the indicated time points.

### Flow cytometry

Phenotype of human B cells was determined with the following antibodies: CD19-V500, CD40-PE-Cy5, CD27-BV421, CD95-AF647, IgD-PE-Cy7, TRAILR2 PE. Dead cell exclusion was performed by Live/Dead staining kit (Invitrogen, Molecular Probes, Carlsbad, CA, USA), following the manufacturer instruction. B-cell proliferation was monitored by CFSE (carboxyfluorescein diacetate, succinimidyl ester; Molecular Probes) labelling. Phospho-p65 was determined as follows: 1–2 × 10^5^ magnetically isolated CD27+ B cells were seeded in 100 *μ*l of medium in a 96-well plate, incubated for 1 h, and then stimulated with CD40L for 5 min. Cells were then fixed with Cytofix (Becton Dickinson, Franklin Lakes, NJ, USA) and incubated for 10 min at 37 °C. After washing, cells were stained for surface markers for 15 min in ice. Afterwards the cells were washed and permeabilized by drop-wise addition of 50 *μ*l ice-cold Phosflow Perm Buffer III (Becton Dickinson), gentle vortexing and incubation on ice for 30 min. This was followed by two more washing steps, and staining for intracellular phospho-NF-*κ*B p65 (Ser536). The data were acquired with BD FACS Canto II (Becton Dickinson) and were analyzed with FlowJo software version 8.7 (TreeStar Inc., Ashland, OR, USA).

### Luciferase reporter assay

HEK293T cells were cultured in 96-well plates at 3 × 10^5^ cells/ml in 100 *μ*l. After 24 h, cells were transfected with a mix of vectors containing: EGFP (transfection efficiency control) (7 ng), control Renilla vector (7 ng), NF-*κ*B firefly luciferase reporter vector (7 ng), CD40 (0.5 ng), CD40L (1 ng) and increasing concentrations of GPI-anchored CD40, TRAILR2, Fas or TACI (0 to 34 ng) (70 ng per well total DNA), using Polyfect transfection reagent (Qiagen, Hilden, Germany). After 24 h, cells were lysed and expression of firefly and renilla luciferases was detected with the dual luciferase assay detection kit (Promega, Madison, MI, USA).

### Statistical analysis

FRET experiments were analyzed using unpaired *t*-test, and when multiple receptors where compared using one-way ANOVA with Tukey's post-test. PLA on BJAB cells was analyzed using unpaired *t*-test. PLA on B cells was analyzed using one-way ANOVA (non parametric Kruskal–Wallis test) with Dunn post-test. NF-*κ*B luciferase reporter assay in BJAB cells was analyzed using one-way ANOVA with Tukey post-test. Differences between marginal zone and switched memory B cells were analyzed using unpaired *t*-test. All analyses were performed using GraphPad Prism version 5.00 for Windows, GraphPad Software, San Diego, CA USA; www.graphpad.com.
