# Supplementary material for: Hetero-oligomerization between the TNF receptor superfamily members CD40, Fas and TRAILR2 modulate CD40 signalling
Source: Cell Death Dis. 2017 Feb 9;8(2):e2601–. doi: 10.1038/cddis.2017.22 (PMC5386471; doi:10.1038/cddis.2017.22)

**Table S1**: List and description of the plasmids used in the study.

| Experiment | Code | Coding for | Tag | Backbone |
| --- | --- | --- | --- | --- |
| FRET | ps015 | - | - | PCR3 |
|  | ps2155 | - | EYFP | PCR3 |
|  | ps2156 | - | ECFP | PCR3 |
|  | ps2967 | EYFP-ECFP fusion | - | pcDNA3.1 zeo |
|  | ps2460 | hCD40 1-260 | EYFP | PCR3 |
|  | ps2461 | hCD40 1-260 | ECFP | PCR3 |
|  | ps2159 | hFas 1-227 | EYFP | PCR3 |
|  | ps2160 | hFas 1-227 | ECFP | PCR3 |
|  | ps3074 | hTRAILR1 1-363 | EYFP | PCR3 |
|  | ps3076 | hTRAILR2 1-327 | EYFP | PCR3 |
|  | ps2948 | hBCMA 4-124 | EYFP | PCR3 |
|  | ps2975 | hBAFFR 1-136 | EYFP | PCR3 |
|  | ps2252 | hTACI 1-223 | EYFP | PCR3 |
|  | ps2253 | hTACI 1-223 | ECFP | PCR3 |
|  | - | ErbB1 | EYFP | Other |
|  | - | ErbB2 | EYFP | Other |
|  | ps3179 | hCD40 1-225 (ΔICD) | EYFP | PCR3 |
|  | ps3180 | hCD40 1-225 ΔICD) | ECFP | PCR3 |
|  | ps3181 | hFas 1-195 (ΔICD) | EYFP | PCR3 |
|  | ps3182 | hFas 1-195 (ΔICD) | ECFP | PCR3 |
|  | ps3309 | hTRAILR2 1-238 (ΔICD) | EYFP | PCR3 |
|  | ps3310 | hTRAILR2 1-238 (ΔICD) | ECFP | PCR3 |
|  | ps3307 | hTACI 1-136 (ΔICD) | EYFP | PCR3 |
|  | ps3320 | hTACI 1-136 (ΔICD) | ECFP | PCR3 |
| NF-kB Luciferase | ps1614 | NF-kB Luciferase | - | Other |
|  | ps1615 | Renilla | - | Other |
|  | ps515 | - | EGFP | pcDNA3.1 zeo |
|  | ps1336 | hCD40 full-length | - | PCR3 |
|  | ps514 | hCD40L full-length | - | PCR3 |
|  | ps687 | hFas 1-170 | GPI | PCR3 |
|  | ps3393 | HA signal hFas 84-170 (ΔCRD1) | GPI | PCR3 |
|  | ps664 | hTRAILR2 1-212 | GPI | PCR3 |
|  | ps3392 | HA signal hTRAILR2 96-212 (ΔCRD1) | GPI | PCR3 |
|  | ps1429 | hCD40 1-193 | GPI | PCR3 |
|  | ps897 | hTACI 2-160 | GPI | PCR3 |
|  | ps1383 | hTACI full-length | - | PCR3 |
|  | ps544 | hBAFF full-length | - | PCR3 |
| FACS | ps864 | ACRP-hBAFF 137-285 | Flag | PCR3 |
|  | ps621 | ACRP-hCD40L 116-261 | Flag | PCR3 |
| CRISPR  Cas9 - KO | ps3373 | TRAILR2 gRNA | - | Addgene 49533 |
|  | ps3374 | CD40 gRNA | - | Addgene 49533 |
|  | ps3375 | Fas gRNA | - | Addgene 49533 |
|  | ps3344 | Pax2 | - | Addgene 12260 |
|  | ps3345 | VSVg | - | Addgene 8454 |


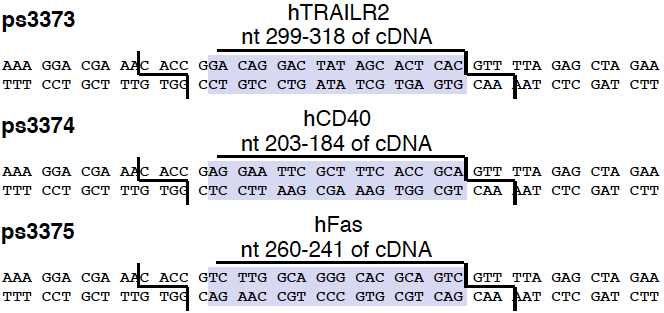


**Supplementary figure 1**

Related to figure 2: Immunostaining and fluorescence intensity plots across a section for CD40 and FAS in BJAB TRAILR2 positive cells. Left panels correspond to the overlay of CD40 (green) and Fas (Red) immunostaining. White squares represent the sections analyzed for fluorescence intensity. Right panels correspond to the fluorescence intensity plots for the indicated regions. Low levels of co-localization can be observed between CD40 and Fas in BJAB TRAILR2 positive cells.


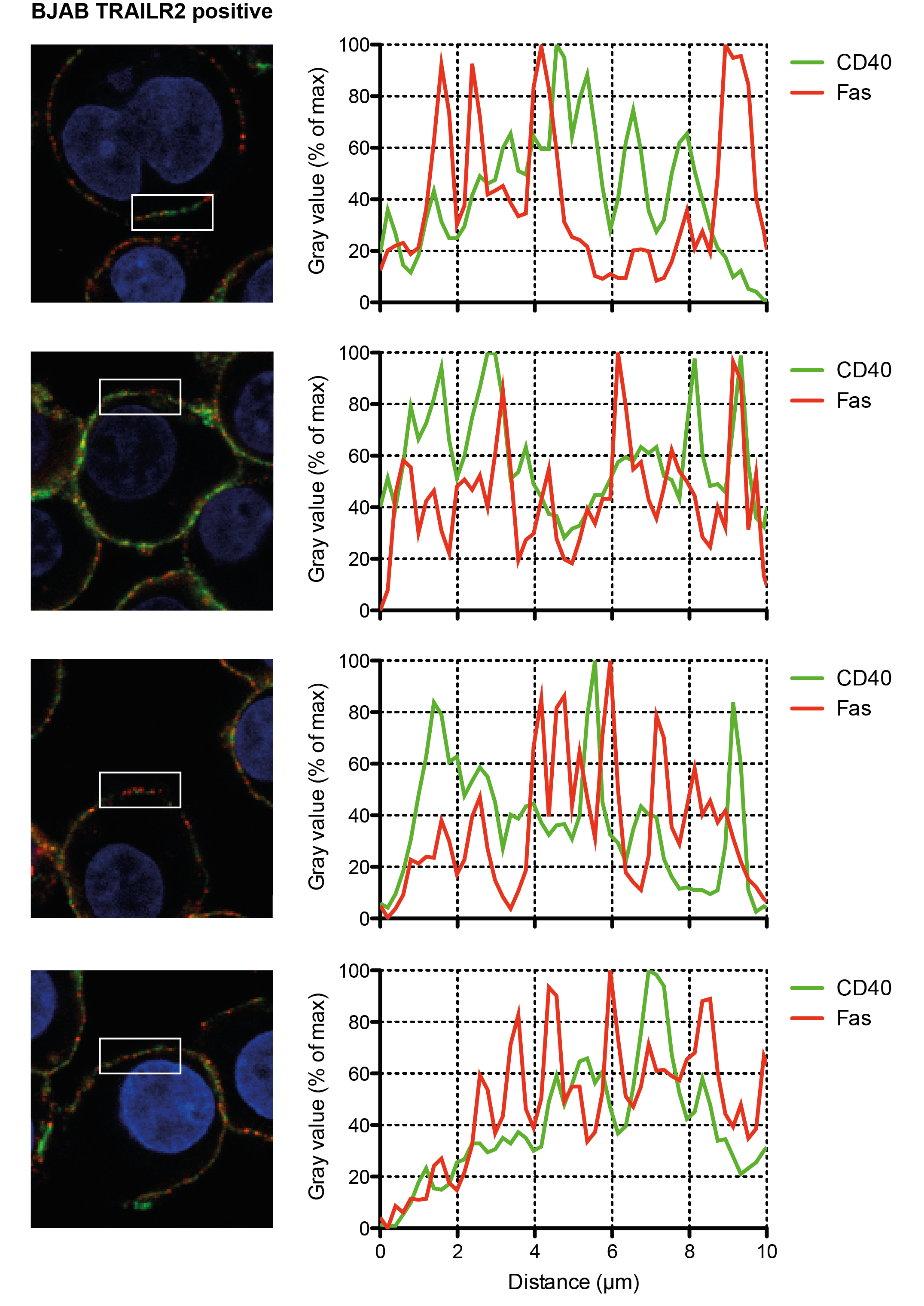


**Supplementary figure 2**

Related to figure 2: Immunostaining and fluorescence intensity plots across a section for CD40 and FAS in BJAB TRAILR2 negative cells. Left panels correspond to the overlay of CD40 (green) and Fas (Red) immunostaining. White squares represent the sections analyzed for fluorescence intensity. Right panels correspond to the fluorescence intensity plots for the indicated regions. High levels of co-localization can be observed between CD40 and Fas in BJAB TRAILR2 negative cells.


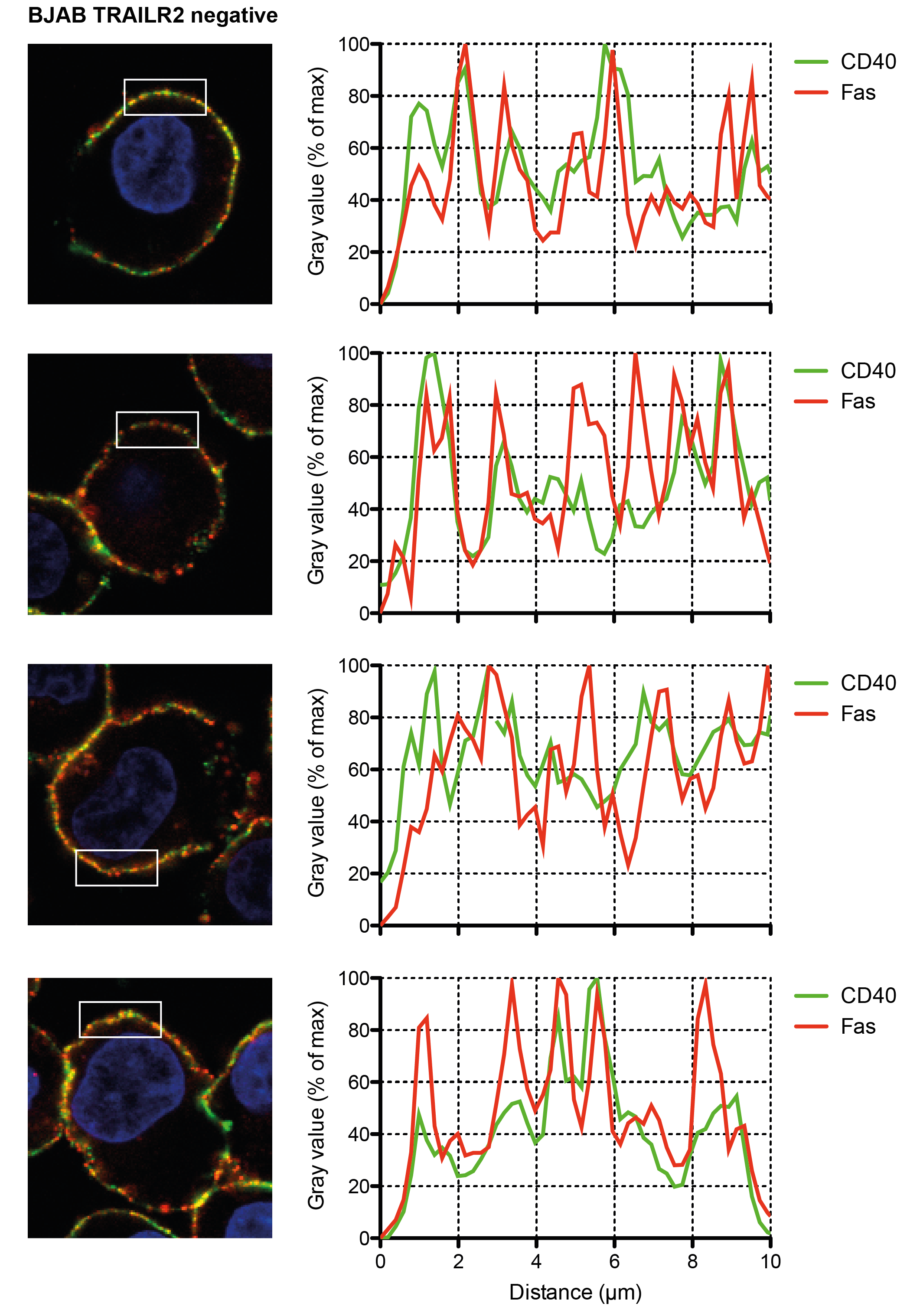


**Supplementary figure 3**

Related to figure 6: Characterization of CRISPR/cas9 KO clones for Fas, CD40 and TRAILR2.

Parental BJAB wild type (wt) cells and selected BJAB clones KO for Fas, CD40 or TRAILR2 where analyzed by flow cytometry for surface expression of Fas (measured by staining with Flag-ACRP-FasL), CD40 (measured by staining with Flag-ACRP-CD40L) or TRAILR2 (measured by staining with anti-TRAILR2 antibody TR2.21). Vertical red lines indicate background staining of controls, green arrows indicate positive staining, and red arrows indicate negative staining in KO clones.


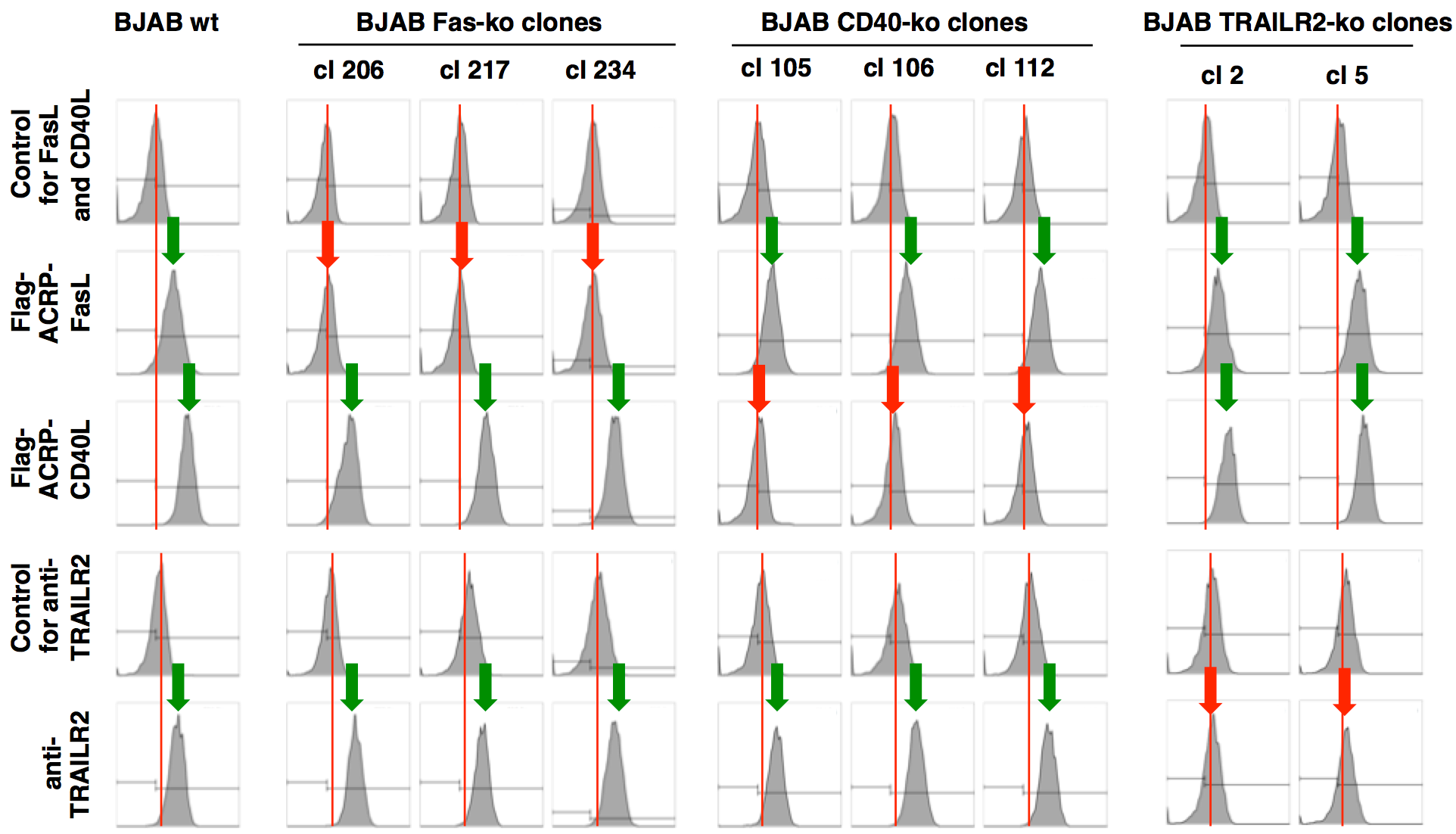

Supplement: Supplementary Information [file cddis201722x1.docx]
